# Fascinating Case Presentation of Moyamoya Disease in Children and Adults

**DOI:** 10.7759/cureus.34081

**Published:** 2023-01-23

**Authors:** Logesh Rajamani, Siddharth Tewari, Rajeswaran Rangasami

**Affiliations:** 1 Radiology, Sri Ramachandra Institute of Higher Education and Research, Chennai, IND

**Keywords:** imaging, moyamoya syndrome, puff of smoke, ivy sign, moyamoya disease, vasculitis

## Abstract

Moyamoya disease is a rare idiopathic disease characterized by progressive stenosis and collateral development of the distal internal carotid arteries. It is mainly seen in East Asia and is the most common cause of stroke in Asian children. However, it is rare in the Indian subcontinent. We present three exciting cases of moyamoya disease with varied clinical presentations in one pediatric, one young adult, and one older patient.

## Introduction

Moyamoya disease (MMD) is a rare progressive idiopathic cerebrovascular disorder characterized by progressive narrowing of the distal internal carotid arteries (ICA) or arteries around the circle of Willis [1]. The development of collaterals is responsible for the characteristic “puff of smoke” appearance at digital subtraction angiography. It is mainly seen in East Asia, especially Japan where the incidence is ~ 1:100,000. Moyamoya disease is considered to be the most common cause of stroke in Asian children [2]. But it is uncommon in the Indian population, especially in children. Moyamoya disease has bimodal age distribution with different clinical presentations in children and adults, as illustrated in the cases below.

## Case presentation

Case 1

**Figure 1:**
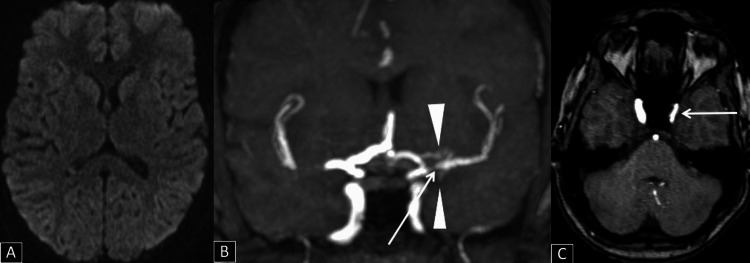
(A) Axial DWI showing no areas of diffusion restriction; (B) Reformatted coronal TOF MRA showing short segment tight stenosis (arrow) in the M1 segment of the left MCA, with few surrounding collaterals (arrowheads) in left MCA cistern; (C) Axial TOF MRA  showing moderate stenosis involving the cavernous segments of the left internal carotid artery (arrow). DWI: Diffusion-weighted image, TOF: Time of flight, MRA: Magnetic resonance angiography, MCA: Middle cerebral artery

A 15-year-old boy presented to the hospital with three episodes of generalized tonic-clonic seizure for one day. The MRI brain showed no evidence of infarct or hemorrhage (Figure 1 A). The magnetic resonance angiography (MRA) showed moderate diffuse stenosis involving the cavernous and supraclinoid segments of the left internal carotid artery (Figure 1 C) and tight stenosis of the M1 segment of the left middle cerebral artery (MCA) with few surrounding collaterals in the left MCA cistern (Figure 1 B). The rest of the intracranial arteries showed no significant pathology. The possibility of moyamoya disease was raised. The child was treated using anti-epileptic medication and was discharged.

Case 2

A 35-year-old man came to the hospital with an episode of generalized tonic-clonic seizure of one-day duration. He had a history of sudden onset hemiplegia involving the left upper and lower limbs two years before presentation due to a large acute right frontal lobe infarct, for which he was admitted to the hospital and received treatment. Since then, he had three other documented episodes of generalized tonic-clonic seizures (one year, three months, and two months prior to presentation). Old imaging reports were unavailable.

**Figure 2:**
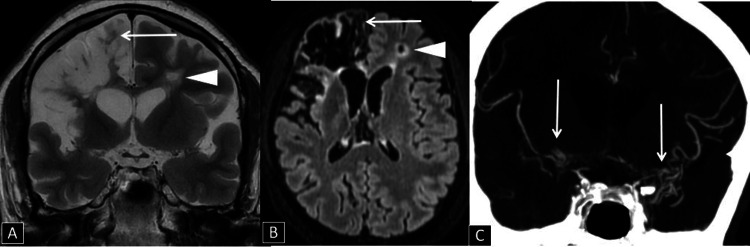
(A) Coronal T2WI image and (B) Axial T2 FLAIR image showing a large area of chronic infarct (arrow) involving the right frontal lobe, right insular region, and right-sided genu of the corpus callosum, and a small chronic infarct (arrowhead) in the left frontal white matter. (C) Coronal reformatted CT Angiogram of the brain showing occluded M1 and M2 segments of the bilateral MCAs replaced by multiple collaterals(arrow)  in the bilateral Sylvian cisterns(more on the left side). T2WI: T2 weighted image, FLAIR: Fluid-attenuated inversion recovery, MCA: Middle cerebral artery

An MRI brain was done, which showed a large area of chronic infarct with gliosis and encephalomalacia involving the right frontal lobe, right insular region, and right-sided genu of the corpus callosum (Figure 2 A and B), and a small chronic infarct in the left frontal white matter (Figure 2 A and B).

A CT Angiogram revealed occlusion of the M1 and M2 segments of the bilateral MCAs with multiple collateral formations around the occluded M1 and M2 segments, particularly on the left side (Figure 2 C). These features are consistent with moyamoya disease. The patient was advised neurosurgical intervention but refused. Currently, the patient is on anti-epileptic medication.

Case 3

A 54-year-old woman was admitted to the hospital with a history of a sudden loss of consciousness for the past six hours. She was unresponsive and came to the hospital with a Glasgow coma scale (GCS) score of 3. She was a known hypertensive and diabetic on medication.

**Figure 3:**
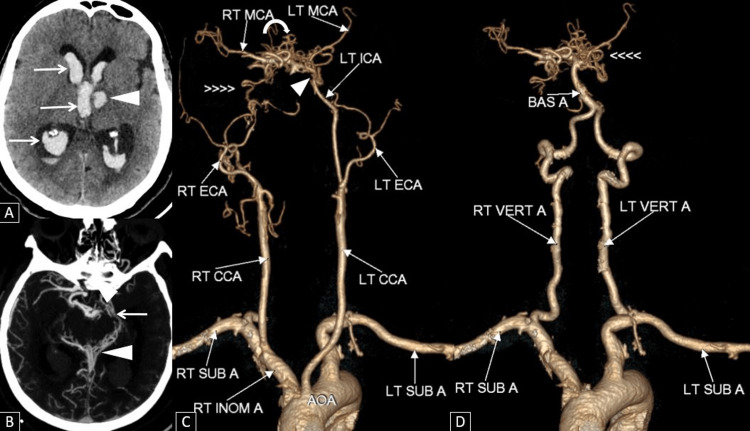
(A) Axial CT brain image showing an acute intraparenchymal hemorrhage in the anterior aspect of the left thalamus (arrowhead) with extensive intraventricular hemorrhage extensions (arrows). (B) Axial reformatted CT angiogram of the brain showing numerous small collaterals in the region of the circle of Willis (arrow) and around the midbrain (arrowhead). (C) Post-processed image of CT angiogram of anterior circulation of the brain showing occluded right internal carotid artery (>>>>), occluded supraclinoid segment of the left internal carotid artery (arrowhead), reformed bilateral MCAs by collaterals (curved arrow) from the vertebrobasilar system. (D) Post-processed image of CT angiogram of posterior circulation of the brain showing mild compensatory dilatation of the basilar artery, and bilateral vertebral arteries with numerous small collaterals in the region of the circle of Willis (<<< LT: Left thalamus, RT: Right thalamus, MCA: Middle cerebral artery, BAS A: Basilar artery, VERT  A: Vertebral arteries, ICA: Internal carotid artery,  CCA: Common carotid artery, ECA: External carotid artery, INOM A: Innominate artery, SUB A: Subclavian artery

On the CT brain, acute intraparenchymal hemorrhage was seen in the anterior aspect of the left thalamus with extensive intraventricular hemorrhage extension into all the ventricles (Figure 3 A). On CT angiogram (Figure 3 B to D), the right internal carotid artery was not visualized, the supraclinoid segment of the left internal carotid artery was occluded, and the bilateral MCAs were reformed by collaterals from the vertebrobasilar system (numerous collaterals are seen in the region of the circle of Willis and around the midbrain with mild compensatory dilatation of the basilar and bilateral vertebral arteries). These findings are consistent with moyamoya disease.

The patient was intubated and kept on mechanical ventilation. Craniotomy was done and an external ventricular drain was placed in the right lateral ventricle. Follow-up CT Brain after four months showed complete resolution of the hemorrhage with residual gliosis and no hydrocephalus, and clinically there was no focal neurological deficit.

## Discussion

Moyamoya disease is primarily an idiopathic disease. It is seen mainly in East Asian countries. Around 10% to 15% of MMD cases may be familial. Male-to-female predominance is 1:1.8, while it is 1:5 for familial cases [1,2]. It is opined to be inherited in an autosomal dominant or polygenic fashion with low penetrance. A recent study showed a high incidence of ring finger protein 213 (RNF213) gene polymorphism (~69%) in patients with MMD compared to patients with vertebral artery dissection [3]. The RNF213 gene polymorphism has been seen in 95% of the familial East Asian population and in 79% of sporadic cases. It is associated with early onset and severe forms of the disease [2,3]. However, MMD-like imaging appearance (termed moyamoya syndrome) can also occur secondary to atherosclerosis, premature aging, or inflammatory states like basal meningitis, central nervous system (CNS) vasculitis, and head and neck infection. It has also been associated with syndromes (such as neurofibromatosis -1, Down syndrome, tuberous sclerosis, Morning Glory syndrome), congenital mesenchymal defects, cranial irradiation in childhood, and prothrombotic states like hereditary spherocytosis and antiphospholipid syndrome. Some authors have shown a link between Epstein-Barr virus infection and MMD [4].

The MMD is characterized by irregular hyperplasia of tunica intima with waving of the internal elastic lamina of affected vessels on histopathology. Thinning of tunica media may result in pseudoaneurysms, later causing hemorrhage. The lumen may collapse resulting in thrombosis leading to ischemic stroke. Although classically described as affecting anterior circulation, over 50% of cases also have involvement in the posterior circulation. This results in diffuse cerebral atrophy, but it tends to mainly affect the regions supplied by anterior circulation. It can be demonstrated on MRI perfusion-weighted imaging where MMD will show reduced perfusion in hemispheric deep white matter with relatively increased perfusion in posterior circulation territory [1-5].

The MMD is commonly seen with bimodal age distribution, with a peak around five to 10 years in children and another peak in the fourth decade in adults. However, clinical presentation differs: whereas adults commonly present with infarcts or intracranial hemorrhage, children mainly will have migraine, seizures, or a history of transient ischemic attack (TIA), as seen in our cases. Moyamoya disease in children can also manifest with poor feeding, developmental delay, and chorea due to brain ischemia but intracranial hemorrhage is rare in children. It has been seen that seizures and epileptiform electroencephalogram (EEG) changes are common in patients with MMD, as seen in our cases. In children, the re-build-up of slow waves at the end of hyperventilation has been said to be characteristic of MMD [2,6]. Hyperventilation-induced reduction in cerebral blood flow may be responsible for the recurrent TIAs seen in pediatric cases, for example when a child is excited, crying, or forcefully blowing on candles. Pediatric MMD has been seen to commonly involve both anterior and posterior circulation compared to adults where mainly anterior circulation involvement is seen.

The MRI brain with contrast and neck and cerebral angiography (MRA) along with a CT angiogram is the common diagnostic tool used to diagnose MMD. The MRA will show classic narrowing of the distal internal carotid artery and/or arteries around the circle of Willis with collaterals. Multiple tiny dot-like flow voids can be noted in the basal ganglia region (due to lenticulostriate and thalamoperforator collaterals) on T2WI and T1WI that enhance on contrast administration [6]. Bright sulci noted on T2 fluid-attenuated inversion recovery (FLAIR) and post-contrast T1WI form the ‘ivy sign’ due to the engorged pial collateral vessels (with slow blood flow) and thickened arachnoid membrane. Dilatation of the medullary veins due to leptomeningeal anastomosis results in a Brush sign (increased conspicuity of dilated medullary veins) on T2* gradient recalled echo (GRE) [1].

An MRI vessel wall imaging can distinguish MMD from moyamoya syndrome. Moyamoya disease typically shows stenosed distal ICA with no significant contrast enhancement without outer wall remodeling as compared to atherosclerosis (outer wall thickening in the stenosed segment compared to the proximal normal segment) or vasculitis (concentric wall thickening showing homogeneous enhancement) [6].

Digital subtraction angiography of cerebral arteries is considered the gold standard [6-7]. Moyamoya disease mainly affects anterior circulation where we can observe narrowed proximal circle of Willis and supraclinoid ICA (earliest sign) followed by the development of lenticulostriate and thalamoperforator collaterals resulting in a characteristic “puff of smoke” appearance. In the late stage, transdural and transosseous collaterals between external carotid and internal carotid branches may be seen. Dilatation of the anterior choroidal artery and its branches is a predictor for intracranial hemorrhage. Although considered to be bilateral, up to 18% of patients with MMD may present with the unilateral angiography-documented disease.

Based on angiography, Suzuki staging was developed in 1969 and is still in use today [1-2]. However, it has been seen to correlate with collateralization in children but not in adults (Table 1).

**Table 1:** Suzuki staging in moyamoya disease ACA: Anterior cerebral artery, MCA: Middle cerebral artery, PCA: Posterior cortical atrophy, ECA: External carotid artery

I	Narrowing of ICA bifurcation
II	ACA, MCA, PCA dilated
III	Intensification of the moyamoya collaterals and narrowed ACA/MCA
IV	Fewer collateral vessels; small PCA
V	Further decrease in collaterals with absent ACA/MCA/PCA
VI	“Disappearance of the moyamoya” - extensive ECA-pial collaterals

The CT brain usually shows diffuse cerebral atrophy (predominantly involving the anterior circulation territory) or may demonstrate the presence of intracranial hemorrhage. The CT angiography demonstrates similar findings as seen on MRA. A Xe-133 CT may show reduced cerebral reserve with acetazolamide challenge [2]

Before making the diagnosis of MMD, it is important to exclude conditions that may lead to moyamoya syndrome. No effective medical treatment for MMD exists yet. Anticoagulation can help to correct/control prothrombotic states.

Surgical intervention is usually required for MMD treatment. Indirect bypass surgery (like encephaloduroarteriosynangiosis) is more commonly performed in children (as their vessels are too small for direct anastomosis), whereas direct bypassing like superficial temporal artery and middle cerebral artery (STA-MCA) is more commonly done in adults [1,8].

## Conclusions

Moyamoya disease has bimodal presentation, commonly presenting with ischemia and stroke in pediatric patients and with hemorrhagic manifestations in adults. It is a chronic pathology and it is important for the neurologist and radiologist to be well-versed in clinical manifestations and imaging features of this condition for early diagnosis and management.
